# Wearable Electronics Assess the Effectiveness of Transcranial Direct Current Stimulation on Balance and Gait in Parkinson’s Disease Patients

**DOI:** 10.3390/s19245465

**Published:** 2019-12-11

**Authors:** Mariachiara Ricci, Giulia Di Lazzaro, Antonio Pisani, Simona Scalise, Mohammad Alwardat, Chiara Salimei, Franco Giannini, Giovanni Saggio

**Affiliations:** 1 Department of Electronic Engineering, University of Rome “Tor Vergata”, 00133 Rome, Italy; rccmch01@uniroma2.it (M.R.); giannini@ing.uniroma2.it (F.G.); 2 Department of Systems Medicine, University of Rome “Tor Vergata”, 00133 Rome, Italy; giulia.dilazzaro@students.uniroma2.eu (G.D.L.); pisani@uniroma2.it (A.P.); simona.scalise@alumni.uniroma2.eu (S.S.); mohammadsamimohammad.alwardat.alwardatmohammad01@alumni.uniroma2.eu (M.A.); chiara.salimei@alumni.uniroma2.eu (C.S.)

**Keywords:** balance, gait, Parkinson’s disease, transcranial direct current stimulation, wearable electronics, IMUs

## Abstract

Currently, clinical evaluation represents the primary outcome measure in Parkinson’s disease (PD). However, clinical evaluation may underscore some subtle motor impairments, hidden from the visual inspection of examiners. Technology-based objective measures are more frequently utilized to assess motor performance and objectively measure motor dysfunction. Gait and balance impairments, frequent complications in later disease stages, are poorly responsive to classic dopamine-replacement therapy. Although recent findings suggest that transcranial direct current stimulation (tDCS) can have a role in improving motor skills, there is scarce evidence for this, especially considering the difficulty to objectively assess motor function. Therefore, we used wearable electronics to measure motor abilities, and further evaluated the gait and balance features of 10 PD patients, before and (three days and one month) after the tDCS. To assess patients’ abilities, we adopted six motor tasks, obtaining 72 meaningful motor features. According to the obtained results, wearable electronics demonstrated to be a valuable tool to measure the treatment response. Meanwhile the improvements from tDCS on gait and balance abilities of PD patients demonstrated to be generally partial and selective.

## 1. Introduction

Wearable electronics are gaining increasing attention and importance as a valid tool for healthcare practitioners in medical treatment [1,2,3] and patient monitoring [4,5,6]. In particular, wearable sensors have been applied for assessing the motor performance of patients with neurodegenerative disorders, as it is for Parkinson’s disease, in both home and clinical environments [7,8,9,10,11,12].

Parkinson’s disease (PD) can be characterized by motor deficiencies, such as bradykinesia and a combination of rest tremor, rigidity, as well as gait and balance impairment [13]. In routine clinical care, the evaluation of those deficiencies is mainly based on severity-rating standardized scales, such as the Movement Disorder Society Unified Parkinson’s disease rating scale (MDS-UPDRS) [14], based on patients’ reports and clinicians’ vision-based evaluations, and clinical investigators determine the effectiveness of a therapy of a drug by using the MDS-UPDRS score [15]. Inconveniently, patient reports can be affected by mood and unfamiliarity with forms, and clinicians’ evaluations can be biased by personal beliefs, experiences, and a priori expectations, resulting in inter- and intra-rater score variability [15,16]. Furthermore, the MDS-UPDRS is quantified according to a discrete scale (0–4, unity step) only, and the human eyes of clinicians hardly detect subtle motor changes during the monitoring of patients. These limitations compel investigators to employ more rigorous, and thus costly, clinical trial designs, with a random assignment of patients, thus blinding investigators to treatment assignment.

The aforementioned limitations can be in some way reduced or overcome through the use of wearable inertial sensors (hereafter wearables), which provide measures of human postures and kinematics, paving the way for objective assessment in clinical trials [17]. In fact, wearables can gather motion parameters in a continuous (analog) or high-step density (digital) scale, and avoid intra- and inter-rater variability, thereby reducing the sample size and simplifying the assessment of the patients, objectively quantifying a possible beneficial effect of a therapeutic intervention. For this reason, even if wearables are still poorly used (only 2.7% of ongoing clinical trials [15]), there is growing attention given to this technological tool, and some pharmaceutical companies are working to develop their own devices [18,19,20].

Our work approaches the utilization of wearables in the particular case of objectively demonstrating the therapeutic beneficial effects, if any, of transcranial direct current stimulation (tDCS) treatment on the motor impairments of patients affected by Parkinson’s disease.

The proven appeal of tDCS is evident as it is a non-invasive, inexpensive, painless brain stimulation technique with many clinical and research applications, ranging from the treatment of depression to neurorehabilitation [21,22]. It consists of applying a direct positive (anodal) or negative (cathodal) 1–2 mA current to the scalp. This stimulation supports the depolarization or hyperpolarization of neurons, thus leading them closer to, or farther away from firing, acting on synaptic transmission or synaptic plasticity [21,23]. Further, tDCS has been used alternatively to (or sometimes concurrently with) dopaminergic drug therapy, because the latter can lose its efficacy during the natural course of the disease, in particular regarding its benefit on postural and gait disorders. Gait is now considered a higher level of cognitive function that involves the integration of attention, planning, memory and other motor, perceptual and cognitive processes. In fact, walking and balance constitute a combination of automatic movement processes, afferent information processing, and intentional adjustments that require a delicate balance between various interacting neuronal systems. In PD, to compensate the loss of motor task, cognitive resources as attention and executive function performed by the dorsolateral pre-frontal cortex (DLPFC) plays a critical role in the relief of gait disorder [24]. In addition, previous studies have shown that anodal tDCS stimulation to either the motor area (M1) or dorsolateral prefrontal cortex (DLPFC) had a significant impact on the motor, non-motor, and balance functional outcomes in PD patients. In fact, brain activation patterns in M1 and DLPFC are extremely involved in successful locomotion performance in patients with PD [21,25,26,27]. Further, the effectiveness of tDCS for alleviating gait and postural instability seems promising [28,29,30,31], however, evidence of its benefit remains unclear and controversial [23,32] because different tDCS protocols and target areas of scalp have been considered, leading to conflicting evidence on MDS-UPDRS scores [23,28].

Our work aims to objectively quantify the motor performance improvements, if any, due to tDCS treatment in a population of patients with PD and gait disturbances. To this aim, we used wearables to measure specific motor tasks, and analyzed the related results by means of the standardized response mean (SRM) index, comparing them with those obtained by the clinical evaluation.

## 2. Materials and Methods

### 2.1. Subjects

Ten PD patients (Table 1) with postural and gait disturbances were recruited at Tor Vergata University Hospital, Rome, Italy. Idiopathic PD was diagnosed according to the MDS clinical diagnostic criteria for PD [13], and patients were enrolled at Hoehn & Yahr disease stages between 1.5 and 4, and with MDS-UPDRS III scores related to a gait higher than 1. Exclusion criteria were age (younger than 30 or older than 85), dementia (mini mental status evaluation, MMSE, score < 24 [33]), therapy changes in the last three months, orthopedic comorbidities, other neurological disorders, and therapy with drugs possibly interfering with motor function (e.g., antipsychotics).

This study was conducted in agreement with the ethical principles of the Helsinki declaration. Informed consent was obtained from each participant and ethical approval was obtained by the local committee (RS 190/18). Patients consented to participate and did not change the therapy during the study, from T0 to T2 (Figure 2), in order to minimize any alteration of motor performance due to dopaminergic therapy variations.

### 2.2. Motor Tests

**Figure 1:**
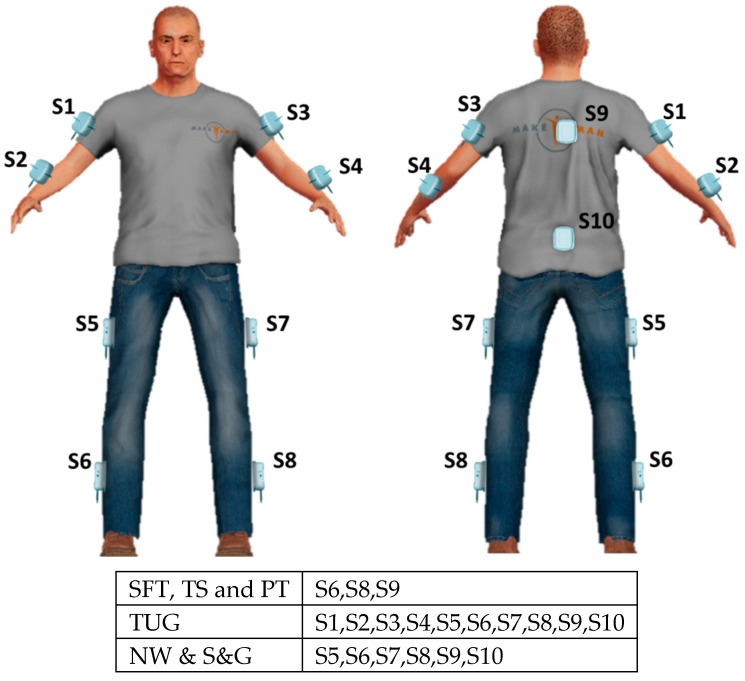
Sensors, labeled from S1 to S10, as located on the body of the patients. Different motor tests resulted with a different number of used sensors.

We requested each participant to perform six motor tasks which, according to clinical standards, are relevant for a comprehensive evaluation of balance and gait. Tasks included stance feet together (SFT), tandem stance (TS), the pull test (PT), timed up and go test (TUG), stop and go test (S&G), and narrow walking test (NW). In particular, SFT and TS are useful to test balance; PT corresponds to the item 3.12 of MDS-UPDRS III to test postural response; TUG, S&G and NW are used to assess mobility and gait. Wearables were placed by means of Velcro strips on segments of the body, according to the particular test, as schematized in Figure 1. The descriptions of the tests and corresponding placements of the wearable sensors are specified in the following.

#### 2.2.1. Stance Feet Together (SFT) and Tandem Stance (TS)

In SFT and TS tests, the patient has to stand and maintain the posture for 30 s. More particularly, in the SFT with feet side-by-side and close together, in TS with feet in tandem position (i.e., one ahead, aligned and close to the other). The wearables were placed on the posterior trunk at the level of T5 and on the external parts of the calf segments of both legs.

#### 2.2.2. Pull Test (PT)

The subject, comfortably standing upright with shoulders to the examiner, is rapidly and vigorously pushed backward on his/her shoulders so as to be forced to make one, or more, steps backwards, recovering his/her balance. The sensors were placed as for SFT and TS.

#### 2.2.3. Timed Up and Go (TUG)

The subject starts seated on a straight-backed chair with arms across the chest, then gets up, walks straight 6 m, turns around, walks straight back and, turning on his/her-self, sits down returning to the initial condition. The sensors were placed on the patient’s pelvis at the level of L5, posterior trunk at the level of T5, on the external parts of thighs and calf segments of both lower limbs, arms, and forearms.

#### 2.2.4. Stop and Go (S&G)

The subject walks for six meters in a straight line, turns around, walks six meters back while the examiner tells him/her to stop and go for 6 times. The sensors were placed on the patient’s pelvis at L5 level, posterior trunk at T5 level, on the external parts of thighs and calf segments of both lower limbs. The time, when the examiner tells the patient to stop was recorded.

#### 2.2.5. Narrow Walking (NW)

The subject walks 6 m straight, but passing through a 70 cm narrow door in the middle of the path. The sensors were placed on the patient’s pelvis at L5 level, posterior trunk at T5 level, on the external parts of thighs, and calf segments of both lower limbs. The time, the time when the patient passes through the door was recorded.

### 2.3. tDCS Stimulation

**Figure 2:**
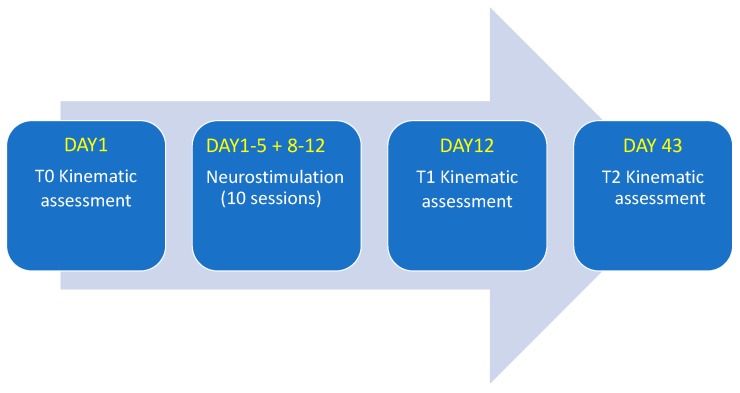
Flow diagram showing the study design and stimulation protocol.

Direct current (DC) was delivered to stimulate the left dorsolateral-prefrontal cortex (DLPFC) by means of a tDCS low-intensity stimulator (BrainStim, EMS Srl, Bologna, Italy). Two saline-soaked electrodes (35 cm^2^) were placed on F4 (according to the 10–20 international EEG nomenclature) and on the right forearm, respectively. The stimulation was of 2mA DC (0.057 mA/cm^2^ in density) delivered for 20 min (30 s step-up ramp, 30 s step-down ramp), repeated ten times, obtaining one session/day, for five consecutive days. Such a stimulation session was followed by two non-stimulation days, and again by another five days of long stimulation (Figure 2). During each tDCS application, patients were at rest without any concurrent motor tasks.

### 2.4. Wearable Electronics

Different technologies can furnish data in terms of gait and balance performances. We can refer, for instance, to pressure sensors embedded into the floor and electro-goniometers, etc., with the optical-based systems considered as the gold standard because of their high accuracy. However, optical-based systems have some important drawbacks, such as the necessities of a free line of sight, time-consuming calibration procedures, necessity of skilled personnel and, above all, a very high cost. Wearable electronics have none of those drawbacks, and have been demonstrated to perform with the appropriate accuracy for our purposes [34,35].

Wearable electronics constitute a network of validated inertial measurement units (IMUs) termed Movit (by Captiks Srl, Rome Italy) [7,34,35], each housing a 3-axis accelerometer (±8 g) and a 3-axis gyroscope (±2000°/s), synchronized to a personal computer receiver, with a 50 Hz data transfer rate. A proprietary application, termed Motion Studio, processes and stores data.

The number of used IMUs and the position of patients’ bodies (by means of elastic bands) varied according to the particular motor tasks performed. Measured data consist of accelerations, angular velocities, and joint angles, computed from the related quaternions via Euler decomposition. In turn, the quaternions are generated using a Kalman filter on data coming from the accelerometers and the gyroscopes, sampled at 200 Hz. By means of a patented calibration procedure, the spatial orientations of the dressed IMUs are represented on a computer screen as a human avatar, which replicates patient movements, with his/her joint angles gathered with a forward kinematic procedure in a parent-child hierarchy.

### 2.5. Features

For each task, we obtained several features, as reported in Table 2 and described in the following paragraphs.

#### 2.5.1. Stance Feet Together (SFT) and Tandem Stance (TS)

Eleven features from the sensor located on the trunk were taken into consideration: range of accelerations, angular velocities and angles of the trunk in the medial-lateral (ML), anterior-posterior (AP) and vertical (V) directions; Jerk and Sway Area. In particular, Jerk, gathered from the accelerometers, represents the time derivative of acceleration [36], and is used as an empirical measure of the smoothness of the movements [37,38]. The Sway Area is the area of the ellipse that encompasses 95% of the values of medial lateral and anterior posterior accelerations around their mean values.

#### 2.5.2. Pull Test (PT)

The PT test is useful to evaluate the postural responses to an unexpected external perturbation. We extracted the 11 features as for the SFT, plus the number of steps following the pushing as resulted from data gathered by the sensors placed on the ankles.

#### 2.5.3. Time Up and Go (TUG)

TUG is one of the most widely used clinical tests and allows for the assessment of several aspects of gait. Parkinsonian gait is characterized by a slowed speed, decreased arm swing, shuffling steps, and difficulty to turn [39]. TUG is composed by four phases: the sit-to-stand phase (patient gets up from the sitting position with arms across the chest), the walking phase (patient walks for 6 m forth and back), the turning phase (the patient turns 180°), and the turn-to-sit phase (the patient turns and sit back on the chair). Each phase is segmented considering data gathered by the IMU on the trunk. We detected the sit-to-stand and turn-to-sit phases considering the interval between the two local minimum values before and after a local maximum of the accelerometer data, in the AP direction, corresponding to the flexion/extension movement of trunk. The turning phase is identified using thresholds on the trunk angle in the vertical direction (the turning component looks as a positive or negative ramp, depending on the direction of the turn). Further details on the segmentation of TUG test are reported in [7].

From these segmentations, 24 features were computer, as described in Table 2, including:

1. Temporal gait characteristics, such as number of steps, step duration, stance duration and swing duration;
2. Features related to upper and lower limb movements, such as the range of motion of arms and legs (Flex Arm, Flex Leg), the average angular velocity (Average Vel) of arms, forearms, legs and thighs, and the asymmetry between right and left limbs (Asym Arm, Asym Leg);
3. Turning parameters, such as the angular velocity of the trunk (Peak Turning Vel), the turning velocity (Turning Vel) and the number of steps (Steps Turning).

#### 2.5.4. Stop and Go (S&G) & Narrow Walking (NW)

Parkinsonian gait problems are often triggered by some circumstances such as spaces with a narrow passage (e.g., a door), unexpected visual or auditory stimuli, stressful situations, cognitive load anxiety and difficulty in starting and stopping [39]. The results are a decreasing step length and step time, decreasing velocity, and increasing variability of step length and time [40,41]. The S&G and NW tests are used to provide evidence for these symptoms. We computed seven features for each task.

For the S&G test, we computed the duration of steps, stance and swing, as well as the angular velocity of the leg of the first steps at the beginning of gait, thus, after each stop signal of the examiner and the variability of the temporal step variables (CV Step, CV Stance, CV Swing).

For the NW test, we computed the same features but extracted them during the 3 s when the patient was passing through the door.

### 2.6. Clinical and Wearables-Based Evaluations

Motor test performances of each of the ten PD patients just before the stimulation protocol (T0 time), just soon after the protocol (T1 time), and 1 month after (T2 time) were evaluated in order to quantify the effect of the tDCS and its persistence, if any.

The evaluations were performed both as standard clinical ones and by the analysis of data gathered through the wearable electronics.

All patients were evaluated by a movement disorder specialist, with general neurological examination, clinical tests, and questionnaires. Clinical tests consisted in the administration of MDS unified Parkinson’s disease rating scale (MDS-UPDRS) and the Berg balance scale (BBS) [43], a clinical five-point ordinal scale that assess balance. Each patient was also evaluated with the freezing of gait questionnaire (FOG-Q) [44], a 6-item questionnaire used to assess gait disturbance severity in patients with PD, and the Hoehn and Yahr scale (H&Y) [45], a commonly used system for describing the progress of symptoms.

To evaluate the responsiveness of a treatment, we considered two aspects. First, we assessed the ability of wearable features to detect change over a particular time frame. Then, we evaluated the relationship between a change in the feature values and the external measure (e.g., the clinical score).

The standardized response mean (SRM) [46] was used to assess the responsiveness to the tDCS therapy. A reason for choosing SRM is because, differently from the paired t-test, it has no dependence on sample size [47]. The SRM expresses the ratio of TT:SDC, where TT is the mean change between T1 and T0 and between T2 and T1, and SDC the standard deviation of the change. Empirically, an SRM value of 0.20 represents a small, 0.50 a moderate, and 0.80 a large responsiveness, respectively.

We used Spearman’s rank correlation coefficient to investigate the relation between the clinical scores and the features. Stance feet together (SFT) and tandem stance (TS) tasks were used to evaluate the static balance, assessed by the clinicians using the BBS scale. Features extracted from SFT and TS are compared with the BBS score. PT features were correlated to the corresponding UPDRS III item 3.12 score (PT is part of UPDRS III tasks). Features extracted from gait related tasks (TUG; ST and NW) were correlated with the UPDRS III gait item score (3.10). The significance level was set at 0.05.

## 3. Results

Table 3 shows the mean, standard deviation values, and SRM of the clinical evaluation results. Table 4, Table 5, Table 6, Table 7, Table 8 and Table 9 report the motor features of SFT, TS, PT, TUG, S&G and NW tests, and correlation analysis between the features and the corresponding clinical evaluation.

### 3.1. Clinical Evaluation

MDS-UPDRS sections two and three, BBS, and FOG-Q (Table 3) demonstrated moderate responsiveness to tDCS at the end of the treatment. The effect appears stable after one month with some improvement in BBS and MDS-UPDRS Section 2 score.

### 3.2. Stance Feet Together (SFT) and Tandem Stance (TS)

Jerk demonstrated a decrement, but only in a small percentage, in SFT (Table 4) and TS (Table 5) in both T1 and T2. During TS, Sway Area, range of the accelerations and angular velocities in the three directions decreased in T1 with a responsiveness around 0.4. The effect is stable at T2 compared to T1 with low improvements in some features.

The BBS score correlates significantly with almost all the features extracted from SFT and TS such as Jerk, Sway area (only TS, r = −0.37) and range of the accelerations and angular velocities. So, features highly reflect the clinical evaluation in this case.

### 3.3. Pull Test (PT)

During the PT, the obtained results (Table 6) showed an unchanged number of steps after tDCS treatment, a small increment of Jerk, and a small reduction of Sway Area at the end of the treatment and one month after.

Regarding the clinical evaluation, only few features (Range Acc V, r = −0.45; Range Gyr ML, r = −0.47) correlated with the UPDRS PT sub score.

### 3.4. Time Up and Go (TUG)

It was found that tDCS showed a moderate effect on the duration of sit-to-stand and walking phase in T1 and T2, as compared to the baseline (Table 7). A lower duration of the Turning phase is present only at T2. In correlation with a lower duration of the walking phase, our results show a reduction of the number of steps and stance duration. No changes were found in features related to the upper limbs. Conversely, the velocity of the lower extremities meaningfully increased. Finally, patients increased the velocity to turn and sit at T1 and T2, with comparison to the baseline values.

The UPDRS gait item score correlates significantly with several features extracted from TUG. Significant correlations regard the features representing the duration of the TUG phases (namely tug time, walk time and turning time). So, patients that take time to complete TUG have higher score on gait item. Weak correlation was for the temporal gait characteristics with the exception of number of steps and CV step. Gait item correlates significantly with features related to lower limb movements (Flex Leg, Average Vel Thigh, and Average Vel Leg) and the turning phase (Turning Vel, Steps Turning).

### 3.5. Stop and Go (S&G) & Narrow Walking (NW)

Both S&G (Table 8) and NW (Table 9) tests show a shorter duration of the step and swing phase and decreased variability of step duration in both T1 and T2 with respect to the baseline. The velocity remained unchanged in S&G but increased in NW. Large responsiveness is found in NW related to step duration, swing duration, velocity, and all the temporal step variability features.

One feature from S&G (step duration, r = −0.42) and two features from NW (Step Velocity, r = −0.44; CV Swing, r = 0.35) are significantly related to the UPDRS gait item.

## 4. Discussion

The response to dopaminergic drug replacement therapy in PD may lose its effectiveness during the course of the disease. Postural and gait disturbances, in particular, are symptoms that are difficult to treat with currently available pharmacological therapies.

Recent studies suggest a potential positive impact of tDCS on gait and balance in PD patients, symptoms of the late stage of PD, poorly responding to the classic dopaminergic treatment.

Our work focused on objectively quantifying the effect of tDCS on gait and postural stability from measured data gathered by wearable electronics used during motor tests of Parkinson’s disease patients.

Within this context, the obtained results demonstrate the impact of wearable electronics with respect to standard clinical evaluation, allowing for interesting insights on the range of change on motor performance following the therapy. In fact, wearable electronics can evidence key elements of postural instability or gait abnormalities, both for evaluating the progression in PD and even to identify the disease at early stages [7,48,49,50]. Accordingly, in this study, specific motor tests were considered to assess the effects of tDCS therapy on balance and gait disturbances, taking into account the effects on measured motor features, soon after the delivery and one month later.

For balance assessment, three different motor tests were adopted to evaluate the equilibrium in three different conditions: SFT for static balance, TS to assess the balance when a low perturbation is introduced, and PT to assess postural responses to an unexpected perturbation. According to the kinematic assessment, Jerk is the only feature that presents a significant variation in SFT, TS and PT, suggesting that it is a highly sensitive measure of balance. This confirms the finding reported in previous studies, wherein Jerk was suggested as a valid biomarker of PD [7,49].

For gait assessment, the TUG test was useful to evaluate the slower speed, decreased arm swing, shuffling steps and difficulty to turn. Further S&G and NW tests were useful to evaluate step time, velocity, and variability of steps, due to the difficulty to start/stop and pass through a narrow door.

Our results show a reduction of step and stance duration and an increment of lower limb velocity during TUG, S&G and NW tests. These achievements confirm the findings reported in other works, which evidenced some improvement of hypokinetic gait in PD after tDCS treatment [29,30,51]. The effect is more evident in NW test, where we observed a large responsiveness to tDCS. The reason why PD patients tend to decrease step time and velocity when approaching a narrowed space is not completely understood [39], however tDCS in some way improves this aspect. We evidenced an improvement of gait in turning and standing tasks during TUG test too, when patients increased the velocity to turn and sit after the stimulation protocol. In particular, changes in turning are one of the early motor deficiencies in PD, as previously reported [50]. The wearable impact in analyzing this complex motor task is relevant. In fact, clinical evaluation alone demonstrated an amelioration in gait and pull test items but was not able to disclose which features of these two motor functions improved. Being able to thoroughly phenotype patients’ motor performances is crucial to understanding the effect of a therapeutic intervention and to allow for speculation with respect to its dynamics.

In order to provide clinical validity for our approach, we investigated the relation between the clinical scores, given by the examiners, and the measured features. Clinical vs. wearables outcomes demonstrated general significant results (Table 4, Table 5, Table 6, Table 7, Table 8 and Table 9). In particular, a higher correlation was found between features extracted from static balance tasks (SFT and TS) and BBS scores and between TUG features and UPDRS gait item scores.

Not all of the features presented a perfect correlation with clinical rating, and this is also expected since these measures should be more sensitive than clinical scales, mostly due to the fact that clinical examination is based on a rating scale with only a few steps, while wearables produce a density scale with a high number of steps [52]. For example, in the TUG test, the duration of the performance is a significant parameter for both the classical clinical exam and “technology-based assessment”. Conversely, the average velocity of lower limbs was significantly and accurately measured only by the wearable sensors. The same consideration applies for the other features extracted from the balance and gait tests. These results are in accordance with a recent work [7], evidencing that several features extracted by sensors were able to detect subtle abnormalities in early stage PD patients where the corresponding clinical score, obtained by visual examination, was considered normal for the majority of subjects.

It could be argued that a better sensitivity can be clinically irrelevant, detecting differences too small to have a real impact on a patient’s life and functioning. Alternatively, it allows investigators to better phenotype motion alterations and their changes after a therapy, and to objectively measure the benefit from a standard intervention, in view of its customization and relevant optimization.

We are aware of some limitations of the present study. First, tDCS was adopted for patients under other medical treatments that had already been adjusted for the optimal dose. We did not use a test-retest design, thus we cannot exclude variability due to participants’ physical or mental conditions, or to drug response fluctuations. To minimize the effects of the aforementioned limitations, we performed the study at the same time of the day for every patient, and no modification to the therapy was allowed in the three months preceding the study and during its course. The study cannot exclude a placebo effect. Moreover, we performed the experiment on a small sample size. Indeed, further studies, on larger cohorts, are mandatory in order to confirm our findings.

## 5. Conclusions

Our study aimed to demonstrate the advantages of outcomes from technology-based measures in clinical trials. These advantages are particularly important for revealing the effectiveness of tDCS protocols in late stage PD patients. This is because the benefit of tDCS remains unclear and controversial, thus the outcomes from electronic wearables can help the clinical rating of the tDCS effectiveness. In particular, our results provide evidence of the wearable electronic impact, as a complementary tool to the standard clinical evaluation.

The adoption of wearables furnished a number of motor features, some of them with a good correlation with standard clinical assessment, others adding information not evident to human eyes.

Nonetheless, even if wearables can provide motor features for an insight of each patient’s motor performances, they remain rarely adopted in clinical trials. We believe that relevant reasons for this can be ascribed to the lack of an integrated platform that can be easily used by nurses and clinicians, and a lack of regulatory approval and appropriate cost–benefit ratios [15,52]. However, the idea to develop and integrate technologies into the assessment of therapy effectiveness has become so evident that several academic centers and companies have started to bring them to the market.

## Figures and Tables

**Table 1:** Patients’ information.

Age	77.2 ± 6.3 y
Gender	7 M, 3 F
Disease duration	10.37 ± 3.8 y
MDS-UPDRS II	15.6 ± 3.66
MDS-UPDRS III	35.2 ± 5.63
Hoehn & Yahr	2.9 ± 0.16
Levodopa equivalent daily dose	771.7 ± 213.58 mg

**Table 2:** Extracted Features from each motor test.

Task	Feature	Description
SFT, TS, PT	Jerk	Time derivative of acceleration in ML and AP directions [42]
	Sway Area	The ellipse that encompasses 95% of the values of ML and AP acceleration around their mean values [42]
	Range	The range of acceleration and angular velocity signals in all the three directions (6 features in total)
PT	# of Steps	The number of steps performed by the subject following the push
TUG	TUG phases duration	Include TUG time (duration of the entire test), sit-to-stand time, walk time, turning time and turn-to-sit time
	# of Steps	Number of steps during the walking phase.
	Gait metrics	Include mean and coefficient of variation of step duration, stance duration, and swing duration
	Flex Arm, Flex Leg	The angular flexion range of arms and legs
	Asym Arm, Asym Leg	Difference in angular flexion range between the faster and slower arm/leg divided by the larger value (lv%)
	Average Vel	The average angular velocity of arm, forearm and thigh along the medial lateral axis during the walking phase
	Turning Vel	The range of turning (180°) divided by turning time
	Peak Turning Vel	The maximum achieved angular velocity of the trunk rotation in the vertical axis during the turning phase
	Steps Turning	The number of steps during the turning phase
	Average Vel SitStand	The average angular velocity of trunk during sit-to-stand in in the anterior posterior plane
S&G	Gait metrics	Mean and coefficient of variation of duration of step, stance and swing computed on first four steps at the beginning of gait, after each stop signal of the examiner
	Step velocity	The angular velocity of legs computed on first four steps at the beginning of gait, after each stop signal of the examiner
NW	Gait metrics	Mean and coefficient of variation of duration of step, stance and swing computed on the 3 s time with patient passing through the door.
	Step velocity	The angular velocity of legs computed on the 3 s time with the patient passing through the door

**Table 3:** Clinical evaluation.

Clinical Evaluation	T0 Mean ± SD	T1 Mean ± SD	T2 Mean ± SD	SRM (T0 vs. T1)	SRM (T0 vs. T2)	SRM (T1 vs. T2)
MDS-UPDRS II	15.6 ± 3.67	13.9 ± 3.21	14.3 ± 3.23	−0.53	−0.42	0.23
MDS-UPDRS III	35.2 ± 5.64	30.5 ± 6.8	30.4 ± 3.47	−0.67	−1.15	−0.01
Gait item (3.10)	2.20 ± 0.60	1.60 ± 0.49	1.50 ± 0.50	−0.90	−0.90	−0.33
PT item (3.12)	1.80 ± 0.75	1.20 ± 0.75	1.60 ± 0.49	−0.65	−0.23	0.82
FOGQ	13.4 ± 3.69	12.5 ± 3.47	12.4 ± 2.11	−0.62	−0.33	−0.04
BBS	42.3 ± 12.35	47.2 ± 7.97	49.3 ± 6.96	0.79	0.82	0.50

**Table 4:** Stance feet together (SFT): feature values at T0, T1, T2; values of SRM comparing times; correlation with BBS score.

Feature (SFT)	T0 Mean ± SD	T1 Mean ± SD	T2 Mean ± SD	SRM (T0 vs. T1)	SRM (T0 vs. T2)	SRM (T1 vs. T2)	Correlation with BBS
Jerk	0.08 ± 0.03	0.07 ± 0.03	0.07 ± 0.03	−0.22	−0.72	−0.15	−0.38 *
Sway Area	0.32 ± 0.22	0.32 ± 0.3	0.32 ± 0.26	−0.01	−0.01	0.01	−0.22
Range Acc V	0.66 ± 0.53	0.61 ± 0.42	0.5 ± 0.34	−0.13	−0.53	−0.39	−0.60 *
Range Acc ML	0.56 ± 0.16	0.59 ± 0.28	0.59 ± 0.24	0.12	0.18	0.05	−0.46 *
Range Acc AP	0.99 ± 0.35	0.92 ± 0.38	0.9 ± 0.37	−0.14	−0.22	−0.07	−0.17
Range Gyr V	7.76 ± 3.45	10.71 ± 5.77	9.04 ± 5.13	0.53	0.24	−0.28	−0.48 *
Range Gyr ML	11.66 ± 5.78	10.95 ± 6.81	9.88 ± 4.87	−0.08	−0.32	−0.20	−0.55 *
Range Gyr AP	4.55 ± 2.35	5.05 ± 4.05	4.32 ± 2.49	0.13	−0.10	−0.27	−0.44 *

* *p* value < 0.05.

**Table 5:** Tandem stance (TS): features values at T0, T1, T2; values of SRM comparing times; correlation with BBS score.

Feature (TS)	T0 Mean ± SD	T1 Mean ± SD	T2 Mean ± SD	SRM (T0 vs. T1)	SRM (T0 vs. T2)	SRM (T1 vs. T2)	Correlation with BBS
Jerk	0.78 ± 1.32	0.21 ± 0.14	0.42 ± 0.45	−0.41	−0.33	0.44	−0.43 *
Sway Area	3.05 ± 4.44	1 ± 0.75	1.14 ± 1.28	−0.43	−0.41	0.13	−0.37 *
Range Acc V	2.79 ± 2.4	1.42 ± 1.36	1.32 ± 1.23	−0.47	−0.62	−0.11	−0.45 *
Range Acc ML	2.73 ± 2.45	1.73 ± 1.48	2.52 ± 2.45	−0.33	−0.08	0.29	−0.51 *
Range Acc AP	3.04 ± 2.88	1.87 ± 0.82	1.77 ± 1.19	−0.39	−0.43	−0.08	−0.35 *
Range Gyr V	40.01 ± 27.54	26.24 ± 12.28	40.06 ± 41.53	−0.42	0.00	0.35	−0.54 *
Range Gyr ML	58.97 ± 73.46	20.48 ± 13.44	29.28 ± 30.11	−0.47	−0.37	0.29	−0.53 *
Range Gyr AP	27.67 ± 26.98	14.68 ± 10.79	15.18 ± 10.01	−0.42	−0.46	0.04	−0.52 *

* *p* value < 0.05.

**Table 6:** Pull test (PT): feature values at T0, T1, T2; values of SRM comparing times; correlation with UPDRS item 3.12 (PT) score.

Feature (PT)	T0 Mean ± SD	T1 Mean ± SD	T2 Mean ± SD	SRM (T0 vs. T1)	SRM (T0 vs. T2)	SRM (T1 vs. T2)	Correlation with PT Item
Number of Steps	4.5 ± 1.8	4.2 ± 1.25	4.2 ± 2.27	−0.15	−0.09	0.00	−0.10
Jerk	11.03 ± 13.43	13.87 ± 18.82	13.64 ± 13.88	0.28	0.40	−0.02	−0.22
Sway Area	99.28 ± 140.03	83.99 ± 91.55	66.66 ± 63.16	−0.22	−0.38	−0.38	−0.27
Range Acc V	15.05 ± 6.23	14.92 ± 5.95	15.71 ± 5.95	−0.02	0.10	0.16	−0.45 *
Range Acc ML	16.33 ± 8.07	15.46 ± 6.17	15.89 ± 8.59	−0.11	−0.06	0.05	−0.28
Range Acc AP	11.52 ± 6.59	13.85 ± 8.27	12.33 ± 6.14	0.34	0.14	−0.23	−0.23
Range Gyr V	238.32 ± 209.22	246.18 ± 166.59	207.18 ± 104.41	0.08	−0.16	−0.27	−0.32
Range Gyr ML	456.96 ± 241.32	340.59 ± 230.85	402.52 ± 228.77	−0.34	−0.22	0.31	−0.47 *
Range Gyr AP	114.13 ± 111.22	91.72 ± 30.45	80.3 ± 31.42	−0.19	−0.33	−0.27	−0.16

* *p*-value < 0.05.

**Table 7:** TUG: feature values at T0, T1, T2; values of SRM comparing times; correlation with UPDRS item 3.10 (Gait) score.

Feature (TUG)	T0 Mean ± SD	T1 Mean ± SD	T2 Mean ± SD	SRM (T0 vs. T1)	SRM (T0 vs. T2)	SRM (T1 vs. T2)	Correlation with Gait Item
Tug Time	32.19 ± 10.24	28.27 ± 9.7	26.8 ± 6.49	−0.48	−0.93	−0.24	0.55 *
Sit-to-Stand Time	3.03 ± 2.64	1.94 ± 0.99	2.12 ± 0.89	−0.41	−0.39	0.13	0.28
Walk Time	20.58 ± 6.96	17.92 ± 6.02	17.81 ± 4.62	−0.52	−0.78	−0.04	0.56 *
Turning Time	3.9 ± 1.71	3.92 ± 2.24	3.09 ± 0.89	0.01	−0.68	−0.44	0.53 *
Turn-to-Sit Time	4.67 ± 1.66	4.48 ± 1.16	3.78 ± 1.2	−0.11	−0.48	−0.67	0.23
Number of Steps	40.13 ± 10.3	37.78 ± 11.76	38.3 ± 9.38	−0.05	−0.57	−0.21	0.49 *
Step duration	1.17 ± 0.08	1.14 ± 0.11	1.15 ± 0.1	−0.34	−0.24	0.21	0.24
Stance	57.94 ± 3.53	55.9 ± 7.88	57.56 ± 3.31	−0.23	−0.30	0.18	0.20
Swing	42.26 ± 3.62	43.97 ± 7.52	42.44 ± 3.31	0.20	0.20	−0.17	−0.22
CV step	0.07 ± 0.03	0.06 ± 0.03	0.09 ± 0.08	−0.24	0.17	0.54	0.47 *
CV Stance	0.08 ± 0.04	0.36 ± 0.89	0.08 ± 0.03	0.30	−0.09	−0.29	0.12
CV Swing	0.13 ± 0.1	0.11 ± 0.08	0.1 ± 0.04	−0.17	−0.27	−0.08	0.21
Flex Leg	23.41 ± 4.43	23.37 ± 7.39	23.91 ± 6.32	−0.01	0.09	0.10	−0.54 *
Flex Arm	30.38 ± 13.57	28.53 ± 18.85	28.1 ± 14.63	−0.13	−0.22	−0.05	0.28
Asym Leg	12.68 ± 6.62	17.03 ± 20.31	16.22 ± 13.22	0.19	0.22	−0.05	0.20
Asym Arm	40.06 ± 27.2	43.99 ± 22.93	40.81 ± 22.05	0.15	0.04	−0.22	−0.07
Average Vel Thigh	38.08 ± 6.56	43.61 ± 8.07	41.66 ± 7.55	0.62	0.49	−0.31	−0.60 *
Average Vel Leg	72.72 ± 17.24	89.6 ± 17.35	88.24 ± 16.19	0.83	0.83	−0.15	−0.52 *
Average Vel Arm	24.42 ± 12.82	25.41 ± 11.03	23.57 ± 9.24	0.14	−0.10	−0.27	0.11
Average Vel Forearm	38.82 ± 17.6	40.26 ± 21.41	34.79 ± 10.74	0.11	−0.28	−0.34	0.06
Turning Vel	51.82 ± 14.09	58.92 ± 23.8	62.47 ± 14.75	0.32	0.93	0.22	−0.53 *
Peak Turning Vel	91.12 ± 18.8	105.04 ± 30.13	101.76 ± 26.65	0.61	0.60	−0.19	−0.25
Steps Turning	5 ± 1	6.5 ± 3.67	5.6 ± 2.65	0.39	0.31	−0.22	0.48 *
Average Vel Sit Stand	27.27 ± 7.96	34.42 ± 11.1	33.37 ± 10.46	0.93	0.61	−0.09	−0.43 *

* *p*-value < 0.05.

**Table 8:** Stop and go (S&G): feature values at T0, T1, T2; values of SRM comparing times; correlation with UPDRS item 3.10 (gait) score.

Feature (S&G)	T0 Mean ± SD	T1 Mean ± SD	T2 Mean ± SD	SRM (T0 vs. T1)	SRM (T0 vs. T2)	SRM (T1 vs. T2)	Correlation with Gait Item
Step duration	1.44 ± 0.38	1.33 ± 0.29	1.34 ± 0.17	−0.21	−0.31	0.04	−0.42 *
Stance	0.99 ± 0.39	0.91 ± 0.33	0.86 ± 0.21	−0.14	−0.35	−0.13	−0.18
Swing	0.45 ± 0.06	0.42 ± 0.07	0.48 ± 0.08	−0.44	0.34	0.86	−0.22
Step velocity	179.34 ± 46.87	184.93 ± 60.03	174.42 ± 47.18	0.08	−0.10	−0.24	−0.08
CV step	0.15 ± 0.12	0.1 ± 0.06	0.1 ± 0.06	−0.35	−0.34	0.05	0.02
CV Stance	0.31 ± 0.22	0.24 ± 0.18	0.33 ± 0.16	−0.21	0.06	0.48	−0.13
CV Swing	0.17 ± 0.04	0.18 ± 0.05	0.17 ± 0.05	0.13	0.04	−0.14	0.07

* *p*-value < 0.05.

**Table 9:** Narrow walking (NW): feature values at T0, T1, T2; values of SRM comparing times; correlation with UPDRS item 3.10 (gait) score.

Feature (NW)	T0 Mean ± SD	T1 Mean ± SD	T2 Mean ± SD	SRM (T0 vs. T1)	SRM (T0 vs. T2)	SRM (T1 vs. T2)	Correlation with Gait Item
Step duration	1.18 ± 0.09	1.09 ± 0.08	1.11 ± 0.09	−1.60	−0.91	0.77	0.25
Stance	0.65 ± 0.12	0.63 ± 0.08	0.65 ± 0.07	−0.17	0.02	0.49	0.05
Swing	0.5 ± 0.03	0.46 ± 0.05	0.47 ± 0.04	−1.58	−0.90	0.50	−0.01
Step velocity	266.98 ± 40.93	297.96 ± 50.31	284.76 ± 39.79	1.56	0.92	−0.66	−0.44*
CV step	0.1 ± 0.05	0.07 ± 0.03	0.08 ± 0.04	−0.59	−0.23	0.27	−0.02
CV Stance	0.14 ± 0.07	0.12 ± 0.04	0.14 ± 0.08	−0.27	−0.01	0.21	0.04
CV Swing	0.13 ± 0.06	0.09 ± 0.03	0.11 ± 0.04	−0.74	−0.37	0.60	0.35*

* *p*-value < 0.05.
